# Supplementary material for: The Immunogenicity of Glutaraldehyde Inactivated PTx Is Determined by the Quantity of Neutralizing Epitopes
Source: Vaccines (Basel). 2025 Jul 31;13(8):817. doi: 10.3390/vaccines13080817 (PMC12390169; doi:10.3390/vaccines13080817)
Supplement: Supplementary file 1 [file vaccines-13-00817-s001.zip › vaccines-3758373-supplementary.pdf]

Table S1. The number of neutralizing epitopes remaining in S1&S2/3 subunits of different PTd preparations and titer of anti-IgG triggered by those PTd preparations.

**Lot# of aP1**	**Neutralizing epitopes remaining in S1 subunit (%)**	**Neutralizing epitopes remaining in S2/3 subunit (%)**	**Anti-IgG (IU/mL)**
#1	60.4	40.6	569.0
#2	44.2	27.0	576.2
#3	52.3	33.8	639.4
#4	43.4	24.7	970.1
#5	41.9	27.5	741.2
#6	38.6	25.5	857.2
#7	37.9	24.3	549.5
#8	39.0	25.7	246.8
#9	60.5	50.8	703.0
#10	57.9	39.7	529.2
#11	19.3	24.2	557.2
#12	20.0	19.5	388.0
#13	21.5	22.6	482.8
#14	23.1	20.9	268.6
#15	47.3	34.9	708.4
#16	34.8	24.1	738.3
#17	33.3	28.7	558.6
#18	26.7	32.0	188.6
#19	23.7	18.6	187.5
#20	28.8	23.4	158.9
#21	71.2	43.2	904.7
#22	53.0	25.5	1054.8
#23	53.1	24.8	693.9
#24	54.0	22.6	618.7
#25	32.9	15.9	374.3
#26	20.4	12.4	450.8
#27	48.2	21.4	1013.1
#28	55.5	21.2	524.1
#29	38.9	15.8	583.7
#30	25.1	14.3	455.3
#31	47.7	24.3	662.1
#32	36.6	21.9	511.2

#1~#8: The PTd samples of different incubation time;
#9~#32: The PTd samples of varying concentrations of glutaraldehyde.

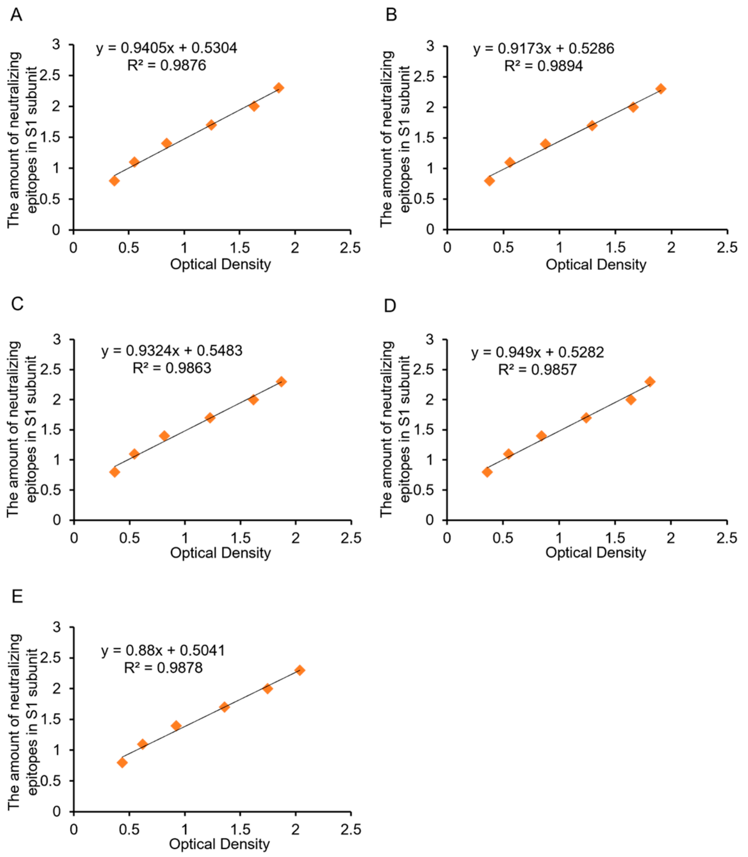

Figure S1. Determination of working range and assay detection limit for the S1 subunit. Both in-house reference and test samples were loaded with concentrations ranging from 6.25 to 200 ng/mL, and dose–response curves were plotted on the number of neutralizing epitopes (Log10) in the S1 subunit. The experiment was repeated 6 times: Experiment# 2 (A); Experiment# 3 (B); Experiment# 4 (C); Experiment# 5 (D); Experiment# 6 (E).

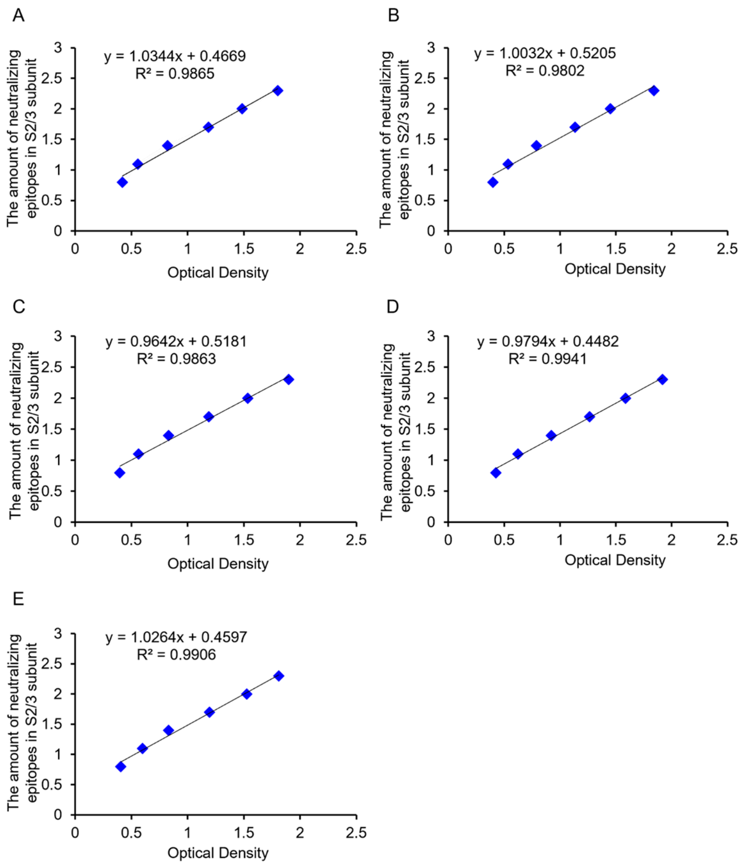

Figure S2. Determination of working range and assay detection limit for the S2/3 subunit. Both in-house reference and test samples were loaded with concentrations ranging from 6.25 to 200 ng/mL, and dose–response curves were plotted on the number of neutralizing epitopes (Log10) in the S2/3 subunit. The experiment was repeated six times: Experiment# 2 (A), Experiment# 3 (B), Experiment# 4 (C), Experiment# 5 (D), and Experiment# 6 (E).

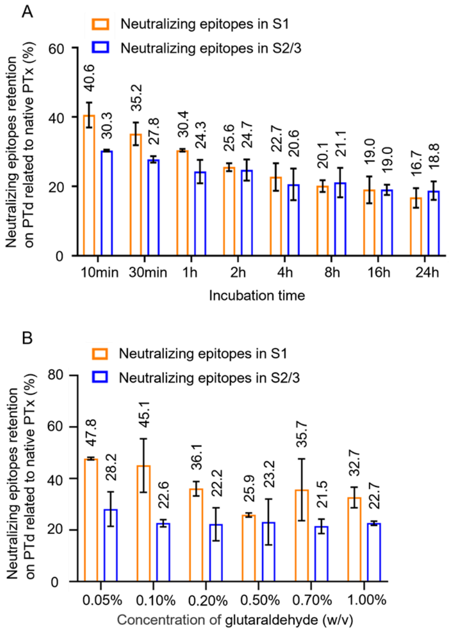

Figure S3. Effect of glutaraldehyde inactivation on neutralizing epitopes in S1 and S2/3 subunits. To study the effect of incubation time on PTx, which had been inactivated by 0.5% (w/v) glutaraldehyde at room temperature (RT) for different times ranging from 10 minutes to 20 hours, to investigate the effect of concentration of glutaraldehyde, the purified PTx was treated with varying concentrations of glutaraldehyde ranging from 0.05% to 1.0% (w/v) at RT for 24 hours; Followingly, neutralizing epitopes retention in S1 and S2/3 subunits of these PTd preparations were determined accordingly (A-B); The retention of neutralizing epitopes on PTd was presented as the percentage (%) related to native PTx.
